# Socioeconomic Inequalities in e-Cigarette Use in Korea: Comparison with Inequalities in Conventional Cigarette Use Using Two National Surveys

**DOI:** 10.3390/ijerph16224458

**Published:** 2019-11-13

**Authors:** Youngs Chang, Sanghyun Cho, Ikhan Kim, Young-Ho Khang

**Affiliations:** 1 Department of Health Policy and Management, Seoul National University College of Medicine, 103 Daehak-ro, Jongno-gu, Seoul 03080, Korea; yschang@snu.ac.kr (Y.C.); jsanghun@gmail.com (S.C.); ikkim85@gmail.com (I.K.); 2 Institute of Health Policy and Management, Seoul National University Medical Research Center, 103 Daehak-ro, Jongno-gu, Seoul 03080, Korea

**Keywords:** diffusion of innovation, electronic nicotine delivery system, smoking, socioeconomic factors, Korea

## Abstract

Socioeconomic inequalities in conventional cigarette smoking are well established in developed countries. The aim of this study was to investigate socioeconomic inequalities in e-cigarette use in Korea, in comparison with inequalities in conventional cigarette use. Data from the Korea National Health and Nutrition Examination Survey (KNHANES) and the Korea Community Health Survey (KCHS) were analyzed. The years of data collected were 2013 to 2016 for the KNHANES and 2014 to 2016 for the KCHS, respectively. The age-adjusted prevalence of ever and current e-cigarette use and conventional cigarette use was calculated according to socioeconomic status indicators, including education, occupation, and income. The prevalence of ever e-cigarette use in men increased from 12.1% in 2013 to 19.2% in 2016 in the KNHANES, and from 13.4% in 2014 to 17.9% in 2016 in the KCHS. Ever and current e-cigarette use was concentrated among current smokers and was much more prevalent among men and also more common among younger age groups in men and women. There was higher prevalences of conventional cigarette use among men and women with less education, manual occupational class and lower income, with the differences more pronounced for women. There was higher ever and current use of e-cigarettes among women with less education, manual occupational class and lower income, but among men there was much less difference in e-cigarette use by these indicators of socio-economic status.

## 1. Introduction

The electronic cigarette (e-cigarette), a device through which the user inhales vapor containing electronically vaporized liquid nicotine, was initially developed in China in the 2000s [1]. E-cigarettes may contain nicotine as well as other chemical substances observed in conventional cigarettes. Previous studies have reported that e-cigarettes, whilst likely to be less hazardous than conventional smoked cigarettes, are very unlikely to be harmless, of particular note, e-cigarette use has been associated with an increased use of conventional cigarettes among youth [2,3,4,5]. Moreover, concerns have been raised regarding the purpose of e-cigarette use, with debates about whether it serves as a replacement for conventional cigarettes, as a tool for smoking cessation, or as a supplement to conventional cigarette smoking [6,7,8,9,10,11,12,13].

The prevalence of e-cigarette use has increased worldwide in recent years. A study conducted in the US showed an increase in the prevalence of ever-use of e-cigarettes among adults from 1.8% in 2010 to 13.0% in 2013 [14]. In a study targeting adults in 27 European Union countries, the prevalence of ever-use of e-cigarettes was found to have increased from 7.2% in 2012 to 11.6% in 2014 [15].

According to the Diffusion of Innovation theory popularized by Everett Rogers, once a new technology is introduced into society, innovators and early adopters adopt it before other members of the community [16]. People of high socioeconomic status tend to be early adopters of new technologies [16,17]. According to the four-stage model of the cigarette epidemic [18,19], people of high social class start to smoke before those of low social class, and they also quit smoking earlier than the latter group. In European countries with an advanced progression of the cigarette epidemic, a more noticeable decrease in the prevalence of conventional cigarette smoking was found among people of high socioeconomic status [20,21,22]. Furthermore, the mortality rate due to smoking was also found to be higher among people of low socioeconomic status [23]. In studies conducted in Korea, it also has been reported that people of low socioeconomic status showed a higher smoking prevalence [24,25,26].

According to a systematic review of the sociodemographic characteristics of e-cigarette users, the prevalence of ever-use of e-cigarettes appeared to be higher among young adults, people of white race, and people with a high level of education [27]. Ooms et al. argued that higher odds of ever-use of e-cigarettes were found among young people and people with a high level of education in 27 European countries [28]. Vardavas et al. also asserted that high odds ratios for the prevalence of ever-use of e-cigarettes were found among young people and metropolitan residents in 27 European countries [29]. However, considering the higher level of education among the younger age group than among the older age group and the decreasing prevalence of cigarette use with age, analyses of the crude prevalence of e-cigarette use by education without adjustment for age may not reveal the role of education in e-cigarette use. The age-adjusted prevalence of e-cigarette use by education level should be examined to investigate the relationship between education and e-cigarette use after accounting for confounding effects of age in the relationship. In a recent study using National Health and Nutrition Examination Survey data from the US, low odds ratios for the prevalence of e-cigarette use were found among people with a high level of education and high income after adjustment for age, sex, and ethnicity [30].

E-cigarette use might be affected by sales areas, the target population for marketing, the population’s attitude toward e-cigarettes, and users’ purpose for using e-cigarettes. Socioeconomic inequalities in e-cigarette use may provide indirect evidence regarding how e-cigarettes are used. According to the Diffusion of Innovation theory [16], it could be assumed that people of high socioeconomic status, who have a high prevalence of smoking cessation, would be likely to be early adopters of e-cigarettes if e-cigarettes are used for smoking cessation. In contrast, it could be expected that using e-cigarettes as a supplement to conventional cigarettes would be reflected by a high prevalence of e-cigarette use among people of low socioeconomic status, in whom conventional cigarette use is common.

Many studies on socioeconomic inequalities in the prevalence of conventional cigarette smoking have been conducted in developed countries, and a high smoking prevalence has generally been found among people of low social class [20,21,22,23]. However, limited studies have investigated socioeconomic inequalities in the prevalence of e-cigarette use on the international level [27,28,29], and no studies have done so using data from Korea. To the best of our knowledge, no previous study has examined socioeconomic inequalities in both e-cigarette use and conventional tobacco use at the same time using nationally representative data. It is important to determine whether e-cigarette use and conventional cigarette use show a similar pattern of inequalities by socioeconomic status. This study examined the distribution of e-cigarette use according to socioeconomic status indicators.

## 2. Materials and Methods

### 2.1. Data

This study used data from two nationally representative surveys: the Korea National Health and Nutrition Examination Survey (KNHANES) [31] and the Korea Community Health Survey (KCHS) [32]. The KNHANES and the KCHS are national surveys conducted annually with a stratified probability sampling method to select representative samples. Both surveys are carried out with face-to-face interview by well-trained interviewers at the survey vehicles for the KNHANES and at the participant’s home for the KCHS. The purpose of the KNHANES is to monitor the health and nutrition status of Koreans. The target sample size of the KNHANES is approximately 13,000 households drawn from about 600 sampling units each year. The purpose of the KCHS is to provide regional health indicators for local district governments to establish and evaluate health policies and programs based on regional health status. The target sample size of the KCHS is approximately 230,000 individuals from about 120,000 households drawn from all of the 252 administrative districts (Si, Gun, Gu) of Korea. Data including e-cigarette related items were collected from 2013 to 2016 from the KNHANES and from 2014 to 2016 from the KCHS. The study population included adults ranging in age from 19 to 64 years, since it was necessary to target the age groups of the economically active population in order to utilize data on occupational class. The total study population was 15,713 from the KNHANES and 490,311 from the KCHS.

### 2.2. Outcome Variables

The study used the prevalence of ever-use and current use of e-cigarettes to calculate the prevalence of e-cigarette use. Ever users of e-cigarettes were defined to be those who responded ‘yes’ to the item “Have you ever used e-cigarettes?” on the KNHANES and the KCHS. Current users of e-cigarettes were defined as individuals who responded ‘yes’ to the item “Have you used e-cigarettes in the last 1 month?”. The prevalence of conventional cigarette smoking was measured by targeting current smokers, who were defined to be those who smoked every day or occasionally among those who reported having smoked more than 5 packs of cigarettes (100 cigarettes) in their lifetime.

### 2.3. Socioeconomic Status

Socioeconomic status indicators included education, occupation, and income. Education was divided into high school or less and college or higher. Occupation was categorized into manual, non-manual, and other occupations using the Korean standard classification of occupations [33]. The category of manual occupations included service workers; sales workers; skilled agricultural, forestry, and fishery workers; craft and related trades workers; equipment, machine-operating, and assembling workers; and unskilled workers. Non-manual occupations included managers and professionals, as well as clerks; other occupations included the armed forces, housewives, and students. The study used annual household income. Equivalized income was calculated by dividing the annual household income by the square root of the number of household members. The equivalized income was divided into tertiles.

### 2.4. Statistical Analysis

Men and women were analyzed separately. Population data from the 2010 Korea Census were used as the standard population for calculating the age-standardized prevalence of ever and current e-cigarette use and conventional cigarette smoking. The age-standardized prevalence was also calculated by socioeconomic status indicators, for which 4 years of data from the KNHANES were combined and analyzed to ensure a sufficient number of smokers. The prevalence of smoking was calculated by applying sample weights. The age-adjusted prevalence ratios (PRs) were computed to measure socioeconomic inequalities in ever and current e-cigarette use and conventional smoking. Using PROC GENMOD, the LINK LOG option in SAS software was employed for PRs [34]. SAS version 9.4 (SAS Institute, Inc., Cary, NC, USA) was used for all statistical analyses.

### 2.5. Ethics Approval and Consent to Participate

This study was approved by the Seoul National University Hospital Institutional Review Board (IRB No. E-1805-047-944). Informed consent for participation in the Korea National Health and Nutrition Examination Survey and the Korea Community Health Survey was granted by all subjects.

## 3. Results

The characteristics of the study subjects are presented in Table 1 and Table 2. The total number of study subjects from the KNHANES was 15,713, excluding 124 respondents with missing information, and 490,311 from the KCHS, excluding 6657 respondents with missing information. In the KNHANES data, men and women aged 19 to 29 years accounted for the largest number of ever and current e-cigarette users, while men and women aged 50 to 64 years accounted for the smallest number of ever and current e-cigarette users. In the KCHS data, the largest number of ever and current e-cigarette users was found among men aged 30 to 39 years and women aged 19 to 29 years. Both the KNHANES and the KCHS data showed that ever and current e-cigarette use was much more prevalent among men and also commoner among younger age groups in men and women. In KNHANES, the prevalence of ever and current e-cigarette use was 15.6% and 4.1% in men, respectively, while the prevalence in women was 1.9% and 0.5%, respectively. The prevalence of e-cigarette use was higher among men aged 19–29 and 30–39 and among women aged 19–29. There was a generally decreasing trend in the prevalence of ever and current e-cigarette use with age among men and women.

In both surveys, the absolute number of male ever and current e-cigarette users was higher among those with a college or higher education than those with a high school or less education. The absolute numbers of ever and current e-cigarette users and conventional cigarette smokers were greater among women with a high school or less education than among those with a college or higher education. In both surveys, the absolute numbers of ever and current e-cigarette users and conventional cigarette smokers in men and women were greater among those with a manual occupation than among those with non-manual occupations. Moreover, the numbers of ever e-cigarette users and conventional cigarette smokers were greater among women with a low income than among those with a high income. In the KCHS data, however, ever and current e-cigarette use was more common among men with a high income, and conventional cigarette smoking was more common among men with a low income.

Table 1 and Table 2 present that most of ever and current users of e-cigarettes were also smoking conventional cigarettes. For example, of total 1205 ever users of e-cigarettes in the KNHANES data, 968 individuals (80.3%) were also currently smoking conventional cigarettes. Of 317 current users of e-cigarettes, 269 subjects (84.9%) were current smokers. Similar findings were noticed in the KCHS data. Of total 33,963 ever users of e-cigarettes, 27,974 subjects (82.3%) were currently smoking conventional cigarettes. Among a total of 6268 current users of e-cigarettes in the KCHS data, 84.5% (5296 subjects) were current smokers. These patterns were generally similar across different age and socioeconomic groups. Table 1 and Table 2 also show that ever and current e-cigarette use was concentrated among current smokers. In the KNHANES data, the percentage of ever and current e-cigarettes users among current smokers was higher than the percentage of never-smokers and ex-smokers. For example, a total of 28.8% (*n* = 968) of total current smokers ever-used e-cigarettes, respectively while only 0.4% (*n* = 35) of total never smokers ever-used e-cigarettes. In the KCHS data, the percentage of ever and current e-cigarettes users among current smokers was also higher than the percentage of never-smokers and ex-smokers. For example, a total of 25.5% (*n* = 27,974) of total current smokers ever-used e-cigarettes, respectively, while only 0.3% (*n* = 782) of total never smokers ever-used e-cigarettes.

Table 3 presents the annual trends in the age-standardized prevalence of ever and current e-cigarette use and conventional cigarette smoking. In the KNHANES, the prevalence of ever e-cigarette use among men rose from 12.1% in 2013 to 19.2% in 2016; in the KCHS, an increase was also found, from 13.4% in 2014 to 17.9% in 2016. Furthermore, in the KNHANES, the prevalence of conventional cigarette smoking among men decreased from 45.5% in 2013 to 40.4% in 2015, and then increased to 42.6% in 2016. In the KCHS, the prevalence of conventional cigarette smoking among men decreased from 45.6% in 2014 to 42.7% in 2016. In the KNHANES, the prevalence of ever and current e-cigarette use and conventional cigarette smoking showed no clear increasing or decreasing trends over time. In the KCHS, however, the prevalence of ever e-cigarette use among women rose from 1.0% in 2014 to 1.6% in 2016, and the prevalence of conventional cigarette smoking among women rose from 3.4% in 2014 to 3.7% in 2016.

Table 4 and Table 5 show the prevalence of ever and current e-cigarette use and the prevalence of conventional cigarette smoking by socioeconomic status. In both surveys, the prevalence of ever and current e-cigarette use and conventional cigarette smoking was higher among men who had a high school or lower education. However, there were no clear differences in prevalence of e-cigarette use by occupational class or income tertile among men in the KNHANES and the KCHS. Meanwhile, in both surveys, a generally high prevalence of ever and current e-cigarette use and conventional cigarette smoking was found among women with a high school or lower education, women working in manual occupations, and women with a low income.

## 4. Discussion

This study investigated annual trends in the prevalence of e-cigarette use and conventional cigarette smoking using nationally representative surveys, the KNHANES and the KCHS. The prevalence of conventional cigarette smoking among men tended to decrease over time; it decreased sharply in 2015 and then increased in 2016. This pattern seems to have been caused by a Korean tobacco control policy that raised the price of a pack of cigarettes from 2500 won to 4500 won (about 4.5 US dollars) in 2015 [35]. The prevalence of ever e-cigarette use increased over time among men and women, mirroring the increase in the prevalence of e-cigarette use that has been observed in other countries [14,15].

Slightly different patterns in inequality by sex and socioeconomic status were found in the prevalence of e-cigarette use and conventional cigarette smoking. The prevalence of ever and current e-cigarette use and conventional cigarette smoking appeared to be higher among men with a high school or lower education. However, the prevalence of e-cigarette use and conventional cigarette use differed by income level among men; men with a low income clearly showed a high prevalence of conventional cigarette use, but the prevalence of e-cigarette use showed a different pattern. The difference in the prevalence of e-cigarette use by income level was found to be unclear, or an opposite pattern was found. Considering the relatively expensive price of e-cigarettes, it is notable that the prevalence of ever and current use of e-cigarettes among men in the high-income group was relatively similar to the prevalence of e-cigarette use among men in the low-income group, whereas the prevalence of conventional cigarette smoking was significantly different according to income groups.

For women, in both the KNHANES and the KCHS, a generally low prevalence of ever and current e-cigarette use and conventional cigarette smoking was found among those with a high socioeconomic status. This pattern held true for education, occupational class, and income. A previous study in the US showed higher odds of current e-cigarette use among people with low education and low income after adjusting for age [30].

An important debate regarding e-cigarettes is whether they are used as an aid for smoking cessation or as a supplement to smoking conventional cigarettes. Quitting conventional cigarette use is more common among people of high social class [20,21,22]. People with high social class are early adopters of new technologies, according to the Diffusion of Innovation theory [16,17]. A high prevalence of e-cigarette use among people with high socioeconomic status would be expected if the main purpose of e-cigarette use is smoking cessation. However, the higher prevalence of e-cigarette among people with a low education level, as well as among women with a low income, as observed in this study, might suggest that those people with low socioeconomic status may have used e-cigarettes as a supplement to conventional cigarette smoking, rather than as aids for smoking cessation. In addition, our analysis findings on ever and current use of e-cigarettes stratified by conventional smoking status (Table 1 and Table 2) showed that most of the ever and current e-cigarette users were concentrated among those who were smoking conventional cigarettes.

In our analysis, the prevalence of ever-use of e-cigarettes in the KCHS data appeared to be slightly lower than the corresponding prevalence in the KNHANES data. This pattern held true for both men and women and held true over time. Among women, particularly, the prevalence of ever-use of e-cigarettes in the KCHS was lower than the prevalence of ever-use of e-cigarettes in the KNHANES data. For example, the prevalence of ever e-cigarette use among men in the KCHS data was 17.9% in 2016, which corresponds to 93% (= 17.9/19.2 × 100) of the prevalence of 19.2% observed in the KNHANES data. In contrast, the prevalence of ever e-cigarette use among women in the KCHS data was 1.6%, which is only 64% (= 1.6/2.5 × 100) of the value of 2.5% from the KNHANES data. This discrepancy most likely reflects differences in how the surveys were conducted; in the KNHANES survey, respondents were interviewed in an independent vehicle used for health examinations [32], whereas the KCHS survey was undertaken by surveyors who visited respondents at home and conducted the survey while the other household members were also present [32]. Thus, the reported prevalence of conventional cigarette smoking among women in the KCHS data was lower than in the KNHANES data. Jung-Choi et al. reported that the prevalence of conventional cigarette smoking among women was more than twice as high when calculated with cotinine concentrations in urine samples from the KNHANES data [36]. In Korea, the social stigma against women smoking might have a major effect on self-reporting, making women less likely to report both e-cigarette use and conventional cigarette smoking, and under-reporting is especially likely when family members are present during the survey. Therefore, the prevalence of e-cigarette use among women in this study, by and large, should be understood to have been under-reported.

This study has strengths. We employed data from large, population-based surveys with multiple measures of socioeconomic status. Two nationally representative data produced generally similar findings on socio-economic differences in e-cigarette use and conventional smoking. This study also has limitations. Our measure for current e-cigarette use was at least monthly use, which would include very occasional users of e-cigarettes. Use of a measure of daily e-cigarette use would be better to assess the extent of regular use of e-cigarettes and explore the degree to which dual use of cigarettes and e-cigarettes was occurring. In addition, we did not have data on reasons for use for e-cigarettes, thus our assumption that high levels of e-cigarette use among smokers with lower socioeconomic status may represent e-cigarette use as a supplement to conventional smoking rather than for cessation is speculative.

## 5. Conclusions

This study examined socioeconomic inequalities in the prevalence of ever and current e-cigarette use and conventional cigarette smoking in Korea using two nationally representative surveys. A high prevalence of conventional cigarette smoking was detected among people of low socioeconomic status—as measured by level of education, occupational status, and income level—and similar patterns were present in men and women. The use of two national survey data produced similar analysis results and thus corroborated the conclusion on the relationship between socioeconomic status and cigarette use. In addition, a high prevalence of e-cigarette use was shown among people with a low level of education. A high prevalence of e-cigarette use was found among low-income women, whereas an unclear pattern was found in the prevalence of e-cigarette use by income level among men. These results indicate that socioeconomic inequalities exist in the prevalence of e-cigarette use, and that these inequalities especially according to education are generally similar to those in the prevalence of conventional cigarette smoking.

## Figures and Tables

**Table 1:** Numbers (*n*) of study subjects, e-cigarette users, and conventional cigarette smokers and their prevalences (95% confidence intervals) by sex according to age groups and socioeconomic status indicators from the Korea National Health and Nutrition Examination Survey (KNHANES) in 2013–2016.

	*n* of Study Subjects	*n* (%) of Ever-Use of E-Cigarettes				*n* (%) of Current Use of E-Cigarettes				*n* (%) of Conventional Cigarette Smokers
		Never Smokers	Ex-Smokers	Current Smokers	Total	Never Smokers	Ex-smokers	Current Smokers	Total	
Total N	15,713	35 (0.4%)	202 (7.3%)	968 (28.8%)	1205 (7.7%, 7.5–7.9%)	7 (0.1%)	41 (1.5%)	269 (8.0%)	317 (2.0%, 1.9–2.1%)	3356 (21.4%, 21.0–21.7%)
Men	6603	13 (0.8%)	167 (7.5%)	849 (30.0%)	1029 (15.6%, 15.1–16.0%)	4 (0.3%)	34 (1.5%)	232 (8.2%)	270 (4.1%, 3.8–4.3%)	2,829 (42.8%, 42.2–43.5%)
Age										
19–29	1138	9 (1.8%)	42 (22.6%)	214 (48.6%)	265 (23.3%, 22.0–24.5%)	2 (0.4%)	4 (2.2%)	66 (15.0%)	72 (6.3%, 5.6–7.0%)	440 (38.7%, 37.2–40.1%)
30–39	1441	0 (0%)	50 (14.8%)	284 (38.0%)	334 (23.2%, 22.1–24.3%)	0 (0%)	14 (4.1%)	69 (9.2%)	83 (5.8%, 5.1–6.4%)	748 (51.9%, 50.6–53.2%)
40–49	1614	1 (0.3%)	33 (5.8%)	198 (26.4%)	232 (14.4%, 13.5–15.2%)	0 (0%)	9 (1.6%)	57 (7.6%)	63 (3.9%, 3.4–4.4%)	750 (46.5%, 45.2–47.7%)
50–64	2410	3 (0.8%)	42 (3.7%)	153 (17.2%)	198 (8.2%, 7.7–8.8%)	2 (0.5%)	7 (0.6%)	40 (4.5%)	49 (2.0%, 1.7–2.3%)	891 (37.0%, 36.0–38.0%)
Education										
High school or less	2828	4 (0.9%)	61 (5.8%)	333 (24.9%)	398 (14.1%, 13.4–14.7%)	2 (0.5%)	11 (1.1%)	102 (7.6%)	115 (4.1%, 3.7–4.4%)	1340 (47.4%, 47.3–47.5%)
College or higher	3775	9 (0.8%)	106 (8.9%)	516 (34.7%)	631 (16.7%, 16.1–17.3%)	2 (0.2%)	23 (1.9%)	130 (8.7%)	155 (4.1%, 3.8–4.4%)	1489 (39.4%, 39.4–39.5%)
Occupational class										
Manual	3148	5 (0.9%)	58 (5.4%)	410 (27.3%)	473 (15.0%, 14.4–15.7%)	1 (0.2%)	12 (1.1%)	105 (7.0%)	118 (3.7%, 3.4–4.1%)	1504 (47.8%, 46.9–48.7%)
Non-manual	2286	1 (0.2%)	72 (8.8%)	295 (33.2%)	368 (16.1%, 15.3–16.9%)	1 (0.2%)	16 (2.0%)	85 (9.6%)	102 (4.5%, 4.0–4.9%)	888 (38.8%, 37.8–39.9%)
Others	1169	7 (1.8%)	37 (10.8%)	144 (33.0%)	188 (16.1%, 15–17.2%)	2 (0.5%)	6 (1.8%)	42 (9.6%)	50 (4.3%, 3.7–4.9%)	437 (37.4%, 36.0–38.8%)
Income tertile										
I (lowest tertile)	2193	6 (1.2%)	58 (8.4%)	284 (28.2%)	348 (15.9%, 15.1–16.6%)	1 (0.2%)	14 (2.0%)	70 (7.0%)	85 (3.9%, 3.5–4.3%)	1,007 (45.9%, 44.9–47.0%)
II	2219	3 (0.6%)	51 (6.7%)	297 (30.8%)	351 (15.8%, 15–16.6%)	3 (0.6%)	9 (1.2%)	79 (8.2%)	91 (4.1%, 3.7–4.5%)	965 (43.5%, 42.4–44.5%)
III (highest tertile)	2191	4 (0.7%)	58 (7.4%)	268 (31.3%)	330 (15.1%, 14.3–15.8%)	0 (0%)	11 (1.4%)	83 (9.7%)	94 (4.3%, 3.9–4.7%)	857 (39.1%, 38.1–40.2%)
Women	9110	22 (0.3%)	35 (6.6%)	119 (22.6%)	176 (1.9%, 1.8–2.1%)	3 (0%)	7 (1.3%)	37 (7.0%)	47 (0.5%, 0.4–0.6%)	527 (5.8%, 5.5–6.0%)
Age										
19–29	1432	7 (0.6%)	21 (16.4%)	48 (38.4%)	76 (5.3%, 4.7–5.9%)	1 (0.1%)	3 (2.3%)	16 (12.8%)	20 (1.4%, 1.1–1.7%)	125 (8.7%, 8.0–9.5%)
30–39	2068	2 (0.1%)	9 (4.3%)	34 (24.3%)	45 (2.2%, 1.9–2.5%)	0 (0%)	2 (1.0%)	13 (9.3%)	15 (0.7%, 0.5–0.9%)	140 (6.8%, 6.2–7.3%)
40–49	2201	5 (0.3%)	2 (2.1%)	23 (17.6%)	30 (1.4%, 1.1–1.6%)	0 (0%)	1 (1.0%)	4 (3.1%)	5 (0.2%, 0.1–0.3%)	131 (6.0%, 5.4–6.5%)
50–64	3409	8 (0.3%)	3 (3.0%)	14 (10.7%)	25 (0.7%, 0.6–0.9%)	2 (0.1%)	1 (1.0%)	4 (3.1%)	7 (0.2%, 0.1–0.3%)	131 (3.8%, 3.5–4.2%)
Education										
High school or less	4851	14 (0.3%)	13 (5.3%)	69 (19.3%)	96 (2.0%, 1.8–2.2%)	2 (0%)	4 (1.6%)	21 (5.9%)	27 (0.6%, 0.4–0.7%)	358 (7.4%, 7.0%–7.8%)
College or higher	4259	8 (0.2%)	22 (7.6%)	50 (29.6%)	80 (1.9%, 1.7–2.1%)	1 (0%)	3 (1.0%)	16 (9.5%)	20 (0.5%, 0.4–0.6%)	169 (4.0%, 3.7–4.3%)
Occupational class										
Manual	2885	10 (0.4%)	6 (4.9%)	50 (22.6%)	66 (2.3%, 2.0–2.6%)	1 (0%)	3 (2.4%)	13 (5.9%)	17 (0.6%, 0.4–0.7%)	221 (7.7%, 7.2–8.2%)
Non-manual	2257	5 (0.2%)	9 (6.7%)	27 (28.1%)	41 (1.8%, 1.5–2.1%)	1 (0%)	1 (0.7%)	10 (10.4%)	12 (0.5%, 0.4–0.7%)	96 (4.3%, 3.8–4.7%)
Others	3968	7 (0.2%)	20 (7.2%)	42 (20.0%)	69 (1.7%, 1.5–1.9%)	1 (0%)	3 (1.1%)	14 (6.7%)	18 (0.5%, 0.3–0.6%)	210 (5.3%, 4.9–5.6%)
Income tertile										
I (lowest tertile)	3020	5 (0.2%)	20 (8.7%)	56 (21.7%)	81 (2.7%, 2.4–3.0%)	1 (0%)	4 (1.7%)	14 (5.4%)	19 (0.6%, 0.5–0.8%)	258 (8.5%, 8.0–9.1%)
II	3065	10 (0.4%)	8 (4.6%)	38 (22.6%)	56 (1.8%, 1.6–2.1%)	1 (0%)	2 (1.1%)	18 (10.7%)	21 (0.7%, 0.5–0.8%)	168 (5.5%, 5.1–5.9%)
III (highest tertile)	3025	7 (0.3%)	7 (5.4%)	25 (24.8%)	39 (1.3%, 1.1–1.5%)	1 (0%)	1 (0.8%)	5 (5.0%)	7 (0.2%, 0.1–0.3%)	101 (3.3%, 3.0–3.7%)

Notes: The total number of never smokers was 1543 for men and 8050 for women, respectively, while the number of ex-smokers was 2231 for men and 533 for women, respectively. The number of current smokers was 2829 for men and 527 for women, respectively. The ‘Total’ columns for the ever- and current use of e-cigarettes presented numbers of ever- and current uses of e-cigarettes and their prevalence (95% confidence intervals) among total study subjects stratified by age group, education, occupational class, and income tertile. For example, of a total of 1138 men aged 19–29, 265 men ever-used e-cigarettes, and the prevalence of ever-use of e-cigarettes among men aged 19–29 was 23.3%. The percentage of the ‘Never smokers’, ‘Ex-smokers’, and ‘Current smokers’ columns referred to the percentage of ever- or current e-cigarette users among each group of never smokers, ex-smokers, and current smokers. For example, among men aged 19–29 who were currently smoking, 214 men ever-used e-cigarettes and the prevalence of ever-use of e-cigarettes among male current smokers aged 19–29 was 48.6%.

**Table 2:** Numbers (*n*) of study subjects, e-cigarette users, and conventional cigarette smokers and their prevalences (95% confidence intervals) by sex according to age groups and socioeconomic status indicators from the Korea Community Health Survey (KCHS) in 2014–2016.

	*n* of Study Subjects	*n* (%) of Ever Use of E-Cigarettes				*n* (%) of Current Use of E-Cigarettes				*n* (%) of Conventional Cigarette Smokers
		Never Smokers	Ex-Smokers	Current Smokers	Total	Never Smokers	Ex-Smokers	Current Smokers	Total	
Total N	490,311	782 (0.3%)	5207 (7.1%)	27,974 (25.5%)	33,963 (6.9%, 6.9%–7.0%)	104 (0%)	868 (1.2%)	5296 (4.8%)	6,268 (1.3%, 1.3–1.3%)	109,791 (22.4%, 22.3–22.5%)
Men	228,963	539 (0.9%)	4754 (7.0%)	26,036 (25.8%)	31,329 (13.7%, 13.6–13.8%)	84 (0.1%)	804 (1.2%)	4889 (4.8%)	5777 (2.5%, 2.5–2.6%)	101,074 (44.1%, 44.0–44.2%)
Age										
19–29	35,078	250 (1.3%)	849 (26.6%)	5870 (44.8%)	6969 (19.9%, 19.7–20.1%)	31 (0.2%)	191 (6.0%)	1223 (9.3%)	1445 (4.1%, 4.0–4.2%)	13,095 (37.3%, 37.1–37.6%)
30–39	45,776	115 (0.9%)	1464 (15.3%)	8306 (35.7%)	9885 (21.6%, 21.4–21.8%)	20 (0.2%)	312 (3.3%)	1693 (7.3%)	2025 (4.4%, 4.3–4.5%)	23,236 (50.8%, 50.5–51.0%)
40–49	59,210	101 (0.9%)	1163 (6.7%)	6623 (22.0%)	7887 (13.3%, 13.2–13.5%)	18 (0.2%)	170 (1.0%)	1176 (3.9%)	1364 (2.3%, 2.2–2.4%)	30,159 (50.9%, 50.7–51.1%)
50–64	88,899	73 (0.4%)	1278 (3.4%)	5237 (15.1%)	6588 (7.4%, 7.3–7.5%)	15 (0.1%)	131 (0.3%)	797 (2.3%)	943 (1.1%, 1.0–1.1%)	34,584 (38.9%, 38.7–39.1%)
Education										
High school or less	111,450	184 (0.9%)	1798 (4.9%)	11,489 (20.9%)	13,471 (12.1%, 12.0–12.2%)	36 (0.2%)	268 (0.7%)	2003 (3.7%)	2307 (2.1%, 2.0–2.1%)	54,863 (49.2%, 49.1–49.4%)
College or higher	117,513	355 (0.9%)	2956 (9.6%)	14,547 (31.5%)	17,858 (15.2%, 15.1–15.3%)	48 (0.1%)	536 (1.7%)	2886 (6.2%)	3470 (3.0%, 2.9–3.0%)	46,211 (39.3%, 39.2–39.5%)
Occupational class										
Manual	125,880	246 (1.0%)	2311 (6.0%)	14,891 (24.0%)	17,448 (13.9%, 13.8–14.0%)	38 (0.2%)	358 (0.9%)	2628 (4.2%)	3,024 (2.4%, 2.4–2.4%)	62,095 (49.3%, 49.2–49.5%)
Non-manual	66,850	134 (0.7%)	1762 (8.5%)	7834 (30.1%)	9730 (14.6%, 14.4–14.7%)	27 (0.1%)	336 (1.6%)	1599 (6.1%)	1,962 (2.9%, 2.9–3.0%)	26,054 (39.0%, 38.8–39.2%)
Others	36,233	159 (1.1%)	681 (7.9%)	3311 (25.6%)	4151 (11.5%, 11.3–11.6%)	19 (0.1%)	110 (1.3%)	662 (5.1%)	791 (2.2%, 2.1–2.3%)	12,925 (35.7%, 35.4–35.9%)
Income tertile										
I (lowest tertile)	76,312	165 (0.8%)	1281 (6.2%)	8229 (22.7%)	9675 (12.7%, 12.6–12.8%)	26 (0.1%)	225 (1.1%)	1508 (4.2%)	1,759 (2.3%, 2.3–2.4%)	36,324 (47.6%, 47.4–47.8%)
II	75,690	172 (0.9%)	1664 (7.2%)	8679 (26.1%)	10,515 (13.9%, 13.8–14.0%)	23 (0.1%)	279 (1.2%)	1631 (4.9%)	1933 (2.6%, 2.5–2.6%)	33,240 (43.9%, 43.7–44.1%)
III (highest tertile)	76,961	202 (1.0%)	1809 (7.5%)	9128 (29.0%)	11,139 (14.5%, 14.3–14.6%)	35 (0.2%)	300 (1.2%)	1750 (5.6%)	2085 (2.7%, 2.7–2.8%)	31,510 (40.9%, 40.8–41.1%)
Women	261,348	243 (0.1%)	453 (7.7%)	1938 (22.2%)	2634 (1.0%, 1.0–1.0%)	20 (0%)	64 (1.1%)	407 (4.7%)	491 (0.2%, 0.2–0.2%)	8717 (3.3%, 3.3–3.4%)
Age										
19–29	39,542	118 (0.3%)	175 (18.9%)	717 (45.2%)	1010 (2.6%, 2.5–2.6%)	8 (0%)	34 (3.7%)	166 (10.5)	208 (0.5%, 0.5–0.6%)	1585 (4.0%, 3.9–4.1%)
30–39	51,473	55 (0.1%)	171 (8.9%)	523 (30.3%)	749 (1.5%, 1.4–1.5%)	2 (0%)	20 (1.0%)	110 (6.4%)	132 (0.3%, 0.2–0.3%)	1725 (3.4%, 3.3–3.4%)
40–49	66,093	36 (0.1%)	53 (3.8%)	344 (15.9%)	433 (0.7%, 0.6–0.7%)	5 (0%)	7 (0.5%)	83 (3.8%)	95 (0.1%, 0.1–0.2%)	2168 (3.3%, 3.2–3.3%)
50–64	104,240	34 (0%)	54 (3.2%)	354 (10.9%)	442 (0.4%, 0.4–0.4%)	5 (0%)	3 (0.2%)	48 (1.5%)	56 (0.1%, 0–0.1%)	3239 (3.1%, 3.1–3.2%)
Education										
High school or less	150,726	89 (0.1%)	239 (6.5%)	1281 (19.2%)	1609 (1.1%, 1.0–1.1%)	9 (0%)	39 (1.1%)	270 (4.0%)	318 (0.2%, 0.2–0.2%)	6684 (4.4%, 4.4–4.5%)
College or higher	110,622	154 (0.1%)	214 (9.7%)	657 (32.3%)	1025 (0.9%, 0.9–1.0%)	11 (0%)	25 (1.1%)	137 (6.7%)	173 (0.2%, 0.1–0.2%)	2033 (1.8%, 1.8–1.9%)
Occupational class										
Manual	98,555	74 (0.1%)	133 (6.5%)	970 (21.8%)	1177 (1.2%, 1.2–1.2%)	9 (0%)	15 (0.7%)	202 (4.5%)	226 (0.2%, 0.2–0.2%)	4443 (4.5%, 4.4–4.5%)
Non-manual	61,944	81 (0.1%)	108 (9.9%)	377 (31.1%)	566 (0.9%, 0.9–1.0%)	4 (0%)	20 (1.8%)	95 (7.8%)	119 (0.2%, 0.2–0.2%)	1212 (1.8%, 1.8–1.9%)
Others	100,849	88 (0.1%%)	212 (7.6%)	591 (19.3%)	891 (0.9%, 0.9–0.9%)	7 (0%)	29 (1.0%)	110 (3.6%)	146 (0.1%, 0.1–0.2%)	3062 (3.0%, 3.0–3.1%)
Income tertile										
I (lowest tertile)	85,558	80 (0.1%)	175 (7.0%)	798 (20.0%)	1053 (1.2%, 1.2–1.3%)	6 (0%)	18 (0.7%)	175 (4.4%)	199 (0.2%, 0.2–0.2%)	3986 (4.7%, 4.6–4.7%)
II	89,408	82 (0.1%)	130 (7.1%)	556 (21.0%)	768 (0.9%, 0.8–0.9%)	10 (0%)	25 (1.4%)	105 (4.0%)	140 (0.2%, 0.1–0.2%)	2642 (3.0%, 2.9–3.0%)
III (highest tertile)	86,382	81 (0.1%)	148 (9.4%)	584 (28.0%)	813 (0.9%, 0.9–1.0%)	4 (0%)	21 (1.3%)	127 (6.1%)	152 (0.2%, 0.2–0.2%)	2089 (2.4%, 2.4–2.5%)

Notes: The number of never smokers was 60,157 for men and 246,727 for women, respectively, while the number of ex-smokers was 67,732 for men and 5904 for women, respectively. The number of current smokers was 101,074 for men and 8717 for women, respectively. The ‘Total’ columns for the ever- and current use of e-cigarettes presented numbers of ever- and current uses of e-cigarettes and their prevalence (95% confidence intervals) among total study subjects stratified by age group, education, occupational class, and income tertile. For example, of a total of 35,078 men aged 19–29, 6969 men ever-used e-cigarettes, and the prevalence of ever-use of e-cigarettes among men aged 19–29 was 19.9%. The percentage of the ‘Never smokers’, ‘Ex-smokers’, and ‘Current smokers’ columns referred to the percentage of ever- or current e-cigarette users among each group of never smokers, ex-smokers, and current smokers. For example, among men aged 19–29 who were currently smoking, 5870 men ever-used e-cigarettes and the prevalence of ever-use of e-cigarettes among male current smokers aged 19–29 was 44.8%.

**Table 3:** Trends in age-adjusted prevalence (95% confidence intervals) of ever-use and current use of e-cigarettes and conventional cigarette use by sex from the Korea National Health and Nutrition Examination Survey (KNHANES) and the Korea Community Health Survey (KCHS).

	KNHANES			KCHS		
Year	Prevalence of Ever-Use of E-Cigarettes	Prevalence of Current Use of E-Cigarettes	Prevalence of Conventional Cigarette Use	Prevalence of Ever-Use of E-Cigarettes	Prevalence of Current Use of E-Cigarettes	Prevalence of Conventional Cigarette Use
**Men**						
2013	12.1 (10.1–14.1)	2.2 (1.4–3.0)	45.5 (42.5–48.6)			
2014	15.1 (13.0–17.2)	4.7 (3.3–6.1)	46.5 (13.5–49.4)	13.4 (13.1–12.8)	2.2 (2.0–2.3)	45.6 (45.1–46.0)
2015	21.6 (19.2–24.0)	7.5 (5.9–9.1)	40.4 (37.5–43.3)	16.3 (16.0–16.7)	4.2 (4.0–4.3)	42.4 (41.9–42.9)
2016	19.2 (17.0–21.4)	4.3 (3.3–5.4)	42.6 (39.7–45.5)	17.9 (17.5–18.2)	3.0 (2.9–3.2)	42.7 (42.2–43.1)
**Women**						
2013	2.5 (1.6–3.4)	0.3 (0.1–0.6)	7.5 (6.1–8.9)			
2014	1.6 (0.9–2.2)	0.4 (0.1–0.6)	5.7 (4.3–7.2)	1.0 (0.9–1.1)	0.1 (0.1–0.2)	3.4 (3.3–3.6)
2015	2.9 (2.0–3.9)	1.3 (0.7–2.0)	5.9 (4.5–7.2)	1.5 (1.3–1.6)	0.3 (0.3–0.4)	3.6 (3.4–3.7)
2016	2.5 (1.7–3.4)	0.4 (0.2–0.7)	6.8 (5.5–8.1)	1.6 (1.5–1.8)	0.3 (0.2–0.3)	3.7 (3.5–3.9)

**Table 4:** Age-adjusted prevalence (%) and prevalence ratio (95% confidence intervals, CI) of ever-use and current use of e-cigarettes and conventional cigarette use by sex according to socioeconomic status indicators from the Korea National Health and Nutrition Examination Survey (KNHANES) in 2013–2016.

Socioeconomic Status Indicators	Ever-Use of E-Cigarettes		Current Use of E-Cigarettes		Conventional Cigarette Use	
	Prevalence (%, 95% CI)	Prevalence Ratio (95% CI)	Prevalence (%, 95% CI)	Prevalence Ratio (95% CI)	Prevalence (%, 95% CI)	Prevalence Ratio (95% CI)
**Men**						
Education						
High school or less	20.6 (18.3–22.8)	1.28 (1.12–1.47)	6.3 (4.8–7.8)	1.60 (1.21–2.12)	54.4 (51.8–56.9)	1.39 (1.30–1.48)
College or higher	15.8 (14.4–17.1)	1 (reference)	3.9 (3.2–4.6)	1 (reference)	38.6 (36.7–40.5)	1 (reference)
Occupational class						
Manual	18.0 (16.3–19.8)	1.05 (0.92–1.21)	4.5 (3.6–5.5)	0.96 (0.73–1.28)	50.2 (48.2–52.3)	1.30 (1.22–1.39)
Non-manual	16.6 (14.7–18.5)	1 (reference)	4.3 (3.3–5.3)	1 (reference)	37.8 (35.5–40.2)	1 (reference)
Others	15.4 (12.4–18.4)	0.88 (0.71–1.08)	3.9 (2.3–5.5)	0.98 (0.67–1.43)	42.7 (38.8–46.6)	1.06 (0.95–1.17)
Income tertile						
I (lowest tertile)	16.7 (15.0–18.5)	1.01 (0.86–1.18)	4.4 (3.4–5.4)	0.95 (0.69–1.31)	46.1 (43.8–48.5)	1.14 (1.06–1.23)
II	17.4 (15.5–19.4)	1.05 (0.90–1.23)	4.8 (3.6–6.0)	1.04 (0.74–1.46)	44.5 (42.0–47.0)	1.10 (1.02–1.19)
III (highest tertile)	16.6 (14.7–18.5)	1 (reference)	4.6 (3.5–5.6)	1 (reference)	40.5 (38.1–42.9)	1 (reference)
**Women**						
Education						
High school or less	4.1 (3.0–5.3)	2.51 (1.73–3.63)	1.2 (0.6–1.9)	2.91 (1.36–6.26)	12.8 (11.0–14.6)	3.47 (2.77–4.34)
College or higher	1.6 (1.2–2.0)	1 (reference)	0.4 (0.2–0.6)	1 (reference)	3.8 (3.2–4.5)	1 (reference)
Occupational class						
Manual	3.9 (2.8–5.0)	2.08 (1.34–3.23)	0.9 (0.4-1.4)	1.91 (0.81–4.49)	10.5 (8.8–12.2)	2.39 (1.84–3.11)
Non-manual	1.7 (1.1–2.3)	1 (reference)	0.5 (0.2–0.8)	1 (reference)	4.1 (3.1–5.0)	1 (reference)
Others	2.1 (1.5–2.7)	1.20 (0.80–1.80)	0.5 (0.2–0.8)	1.04 (0.47–2.33)	6.2 (5.2–7.1)	1.52 (1.17–1.97)
Income tertile						
I (lowest tertile)	3.1 (2.3–3.9)	1.88 (1.22–2.90)	0.7 (0.4–1.0)	2.82 (1.03–7.69)	9.3 (8.0–10.6)	2.25 (1.69–2.99)
II	2.4 (1.7–3.1)	1.43 (0.90–2.25)	0.9 (0.5–1.3)	3.39 (1.26–0.08)	6.1 (5.1–7.2)	1.49 (1.12–1.98)
III (highest tertile)	1.6 (1.1–2.2)	1 (reference)	0.2 (0–0.5)	1 (reference)	4.2 (3.2–5.2)	1 (reference)

**Table 5:** Age-adjusted prevalence (%) and prevalence ratio (95% confidence intervals, CI) of ever-use and current use of e-cigarettes and conventional cigarette use by sex according to socioeconomic status indicators from the Korea Community Health Survey (KCHS) in 2014–2016.

Socioeconomic Status Indicators	Ever-Use of E-Cigarettes		Current Use of E-Cigarettes		Conventional Cigarette Use	
	Prevalence (%, 95% CI)	Prevalence Ratio (95% CI)	Prevalence (%, 95% CI)	Prevalence Ratio (95% CI)	Prevalence (%, 95% CI)	Prevalence Ratio (95% CI)
**Men**						
Education						
High school or less	19.5 (19.1–20.0)	1.28 (1.24–1.31)	3.8 (3.6–4.0)	1.24 (1.16–1.33)	54.7 (54.2–55.2)	1.42 (1.41–1.44)
College or higher	14.9 (14.6–15.1)	1 (reference)	2.9 (2.8–3.0)	1 (reference)	38.2 (37.8–38.5)	1 (reference)
Occupational class						
Manual	18.3 (18.0–18.6)	1.15 (1.11–1.18)	3.4 (3.3–3.6)	0.99 (0.93–1.06)	51.3 (50.9–51.7)	1.35 (1.34–1.37)
Non-manual	15.9 (15.5–16.2)	1 (reference)	3.4 (3.2–3.6)	1 (reference)	38.0 (37.5–38.4)	1 (reference)
Others	12.4 (11.8–13.0)	0.69 (0.66–0.73)	2.6 (2.3–2.9)	0.60 (0.53–0.67)	41.0 (40.2–41.9)	0.96 (0.94–0.98)
Income tertile						
I (lowest tertile)	15.4 (15.0–15.7)	0.95 (0.92–0.97)	3.0 (2.8–3.2)	0.92 (0.85–1.00)	47.4 (47.2–48.2)	1.18 (1.16–1.19)
II	15.8 (15.4–16.1)	0.97 (0.94–1.0)	3.1 (2.9–3.2)	0.95 (0.88–1.02)	43.4 (43.0–43.9)	1.07 (1.05–1.08)
III (highest tertile)	16.2 (15.9–16.6)	1 (reference)	3.2 (3.1–3.4)	1 (reference)	40.7 (40.3–41.2)	1 (reference)
**Women**						
Education						
High school or less	3.2 (3.0–3.4)	3.66 (3.28–4.07)	0.7 (0.6–0.8)	4.24 (3.34–5.38)	7.4 (7.1–7.7)	4.18 (3.90–4.49)
College or higher	0.9 (0.8–0.9)	1 (reference)	0.2 (0.1–0.2)	1 (reference)	1.8 (1.7–1.9)	1 (reference)
Occupational class						
Manual	2.8 (2.6–3.0)	2.62 (2.32–2.97)	0.6 (0.5–0.7)	2.74 (2.09–3.59)	6.7 (6.4–7.0)	2.87 (2.65–3.11)
Non-manual	1.0 (0.9–1.1)	1 (reference)	0.2 (0.2–0.3)	1 (reference)	2.1 (2.0–2.3)	1 (reference)
Others	1.1 (1.0–1.2)	1.11 (0.98–1.25)	0.2 (0.1–0.2)	0.85 (0.64–1.12)	2.9 (2.8–3.1)	1.40 (1.29–1.51)
Income tertile						
I (lowest tertile)	1.8 (1.6–1.9)	1.49 (1.33–1.66)	0.3 (0.3–0.4)	1.46 (1.13–1.90)	5.3 (5.0–5.5)	2.08 (1.94–2.22)
II	1.2 (1.1–1.3)	1.0 (0.89–1.13)	0.2 (0.2–0.3)	0.90 (0.68–1.18)	3.3 (3.1–3.4)	1.28 (1.19–1.38)
III (highest tertile)	1.2 (1.1–1.3)	1 (reference)	0.2 (0.2–0.3)	1 (reference)	2.6 (2.5–2.7)	1 (reference)
